# A novel method for preparing microplastic fibers

**DOI:** 10.1038/srep34519

**Published:** 2016-10-03

**Authors:** Matthew Cole

**Affiliations:** 1 College of Life and Environmental Sciences: Biosciences, Geoffrey Pope Building, University of Exeter, Stocker Road, Exeter EX4 4QD, UK; 2 Plymouth Marine Laboratory, Prospect Place, The Hoe, Plymouth PL1 3DH, UK

## Abstract

Microscopic plastic (microplastic, 0.1 µm–5 mm) is a widespread pollutant impacting upon aquatic ecosystems across the globe. Environmental sampling has revealed synthetic fibers are prevalent in seawater, sediments and biota. However, microplastic fibers are rarely used in laboratory studies as they are unavailable for purchase and existing preparation techniques have limited application. To facilitate the incorporation of environmentally relevant microplastic fibers into future studies, new methods are required. Here, a novel cryotome protocol has been developed. Nylon, polyethylene terephthalate and polypropylene fibers (10–28 μm diameter) were aligned, embedded in water-soluble freezing agent, and sectioned (40–100 μm length) using a cryogenic microtome. Microplastic fibers were prepared to specified lengths (P < 0.05, ANOVA) and proved consistent in size. Fluorescent labelling of Nylon microfibers with Nile Red facilitated imaging. A 24 h feeding experiment confirmed bioavailability of 10 × 40 μm Nylon fibers to brine shrimp (Artemia sp). This protocol provides a consistent method for preparing standardised fibrous microplastics, with widths similar to those observed in the natural environment, which could ultimately lead to a better understanding of the biological and ecological effects of microplastic debris in the environment.

Microplastics (microscopic plastic, 0.1 µm–5 mm) are a prolific anthropogenic pollutant, impinging on terrestrial, freshwater and marine ecosystems across the globe^1^,^2^. Microplastic debris encompasses primary microplastics (e.g. exfoliates in cosmetics)^3^, synthetic fibers and secondary microplastics derived from the photo oxidative breakdown of macroplastics^4^. Ecosystems can become contaminated with microplastics through atmospheric fallout^5^, indiscriminate disposal or mishandling of plastic waste^6^, and incomplete screening of wastewater effluent^7^. It is estimated 269,000 metric tonnes of plastic debris are floating on the surface of the world’s oceans^2^, while coastal and deep sea sediments are also widely contaminated with plastic litter^8^. The concentrations and types of plastic sampled can vary considerably, with spatial and temporal variances attributed to a suite of biotic and abiotic factors, and the sampling method implemented^9^,^10^.

Fibres are among the most prevalent types of microplastic debris observed in the natural environment. These synthetic microfibers are typically manufactured from nylon, polyethylene terephthalate (polyester, PET), or polypropylene (PP)^4^,^9^; their presence is commonly attributed to the release of synthetic fibers from garments during washing^11^, degradation of cigarette butts leading to the release of cellulose acetate fibres^12^, and fragmentation of maritime equipment (e.g. ropes and nets). Owing to their myriad sources, the size of microplastic fibres (MFs) identified in environmental samples is understandably variable. MFs sampled in the western English Channel (UK) with a 333 μm Neuston net presented with widths of 6–175 μm, with an average diameter of 28 μm^13^, and lengths of 250–6250 μm. Fibres of this size were also evident in the Gulf of Maine (US), where sampled fibers ranged from 5–593 μm in width, with a median diameter of 24 μm, and lengths of 164–13.4 mm (Cole *et al.*, unpublished data). Use of finer 50 μm hand nets has revealed microfibers with smaller dimensions in significantly greater quantities in Geoje bay (South Korea)^14^. In the marine environment a number of invertebrates have been observed to ingest fibrous microplastics, including: 100% of wild and farmed blue mussels (*Mytilus edulis*) sampled in Nova Scotia (Canada), with an average load of 62–323 microfibers organism^−1 ^15; 5.8% of the euphausiid *Euphausia pacifica* and 2.6% of the copepod *Neocalanus cristatus* sampled from coastal waters of British Columbia (Canada), in which 68% and 44% respectively of microplastics identified were fibrous^16^; and, 83% of langoustine (*Nephrops norvegicus*) sampled in the Clyde estuary (UK), in which monofilament fibers were observed to accumulate and entangle in the animals’ foreguts^17^. However, the risks MF ingestion might pose to aquatic organisms are currently unclear.

The risks microplastic debris pose to aquatic life, ecological processes and food security is a key consideration for environmental monitoring bodies and legislators^18^,^19^,^20^,^21^,^22^. Microplastics are environmentally persistent^23^, have a propensity for adsorbing toxic persistent organic pollutants^20^, and are bioavailable to a wide range of aquatic biota^24^. Toxicity tests using representative microplastics have revealed microplastics can cause adverse health impacts including: reduced feeding, egg size and hatching success in marine copepods^25^,^26^; reduced egg number, egg size and sperm motility in oysters^27^; reduced feeding, burrowing and energetic reserves in a marine polychaete^28^; and reduced motility, predatory response, hatching success and growth in Eurasian perch^29^. On the contrary, a small number of studies have also highlighted that microplastics can have a relatively limited impact on marine invertebrates with simplistic feeding strategies^30^,^31^. Proven ecological impact is instrumental in promoting new environmental legislation, however it is essential such investigations closely consider the environmental relevance of the plastics used (i.e. concentration and types of plastic tested)^32^. An analysis of published research evaluating uptake, adherence or impacts of microplastics on biota in laboratory studies (2000–2015) reveals that spherical microbeads have been used as representative plastics in the majority of such studies (SI, Table S1). Conversely, synthetic fibers, which are environmentally prevalent, are underrepresented^33^. This environmental-laboratory mismatch largely stems from the availability of microplastics suitable for laboratory use. Microbeads are readily purchased from scientific suppliers allowing researchers to conduct replicable, reproducible and robust experiments with plastics consistent in size, polymer, density, fluorescence and surface charge. While irregularly shaped plastic particles (e.g. PVC, Nylon; 200 µm–5 mm) and pre-production pellets can be obtained through plastic manufacturers, synthetic microscopic fibers (MFs) are not available for purchase. Existing methods for preparing MFs are limited to cutting or cryogenically grinding synthetic cord, resulting in relatively large fibres (>500 μm in length) with a wide size distribution^17^,^34^,^35^,^36^,^37^. These methods have produced MFs suitable for consumption by a number of aquatic detritivores, including sea cucumbers, langoustine, freshwater amphipods, isopods, shore crabs and polychaetes^12^,^17^,^34^,^35^,^36^,^37^. However, the subjective nature of these methods limits reproducibility of test fibres, and the relatively large size of the prepared fibres are too big for smaller invertebrates commonly used in aquatic toxicity testing to consume.

To incorporate environmentally relevant fibrous microplastics into toxicity testing of plastics in the laboratory, it is imperative researchers have replicable and reproducible methods for preparing microscopic plastic fibers (microfibers, MFs) of a size appropriate to their test organism. Here, a novel cryogenic microtome (cryotome) protocol for preparing MFs is described and validated. Prepared MFs were imaged using light and electron microscopy, and the size distribution and consistency of fibers considered. Further, a protocol for fluorescently dying microfibers with Nylon red is trialled. Applicability of prepared MFs for laboratory studies was tested using brine shrimp (*Artemia* sp.). The importance of considering fibers in toxicity testing of microplastics is discussed.

## Results and Discussion

The cryotome protocol was optimised to rapidly produce microscopic plastic fibers of a consistently small size. MFs were cut at pre-determined lengths (40, 70 or 100 μm) with a minimum 1:3 aspect ratio, resulting in a distinctive ‘rod’ shape. Nylon microfibers were fluorescently dyed with Nile Red, and proved bioavailable to suspension feeding *Artemia* nauplii.

**Figure 1:**
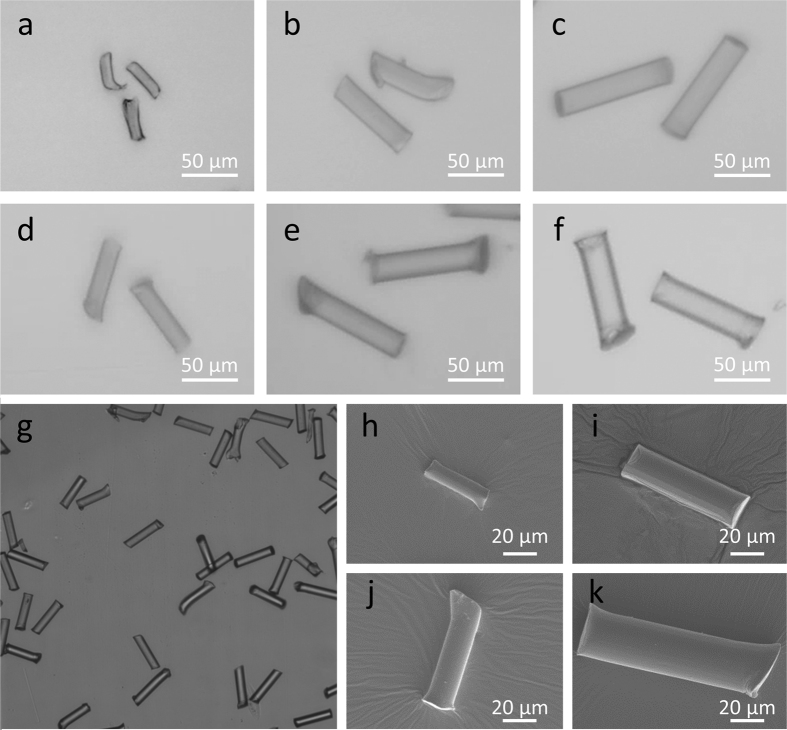
Microscopic fibrous microplastics were prepared using a novel cryotome protocol. Micrographs: (**a**) Nylon 10 × 40 μm; (**b**) Nylon 19 × 70 μm; (**c**) Nylon 23 × 100 μm; (**d**) PET 17 × 70 μm; (**e**) PET 23 × 100 μm; (**f**) PP 28 × 100 μm; (**g**) PET MFs (17 × 70 μm; ×100 magnification); image taken at x10–100 magnification (LEICA DMI 4000). Electron micrographs: (**h**) Nylon 10 × 40 μm; (**i**) fluorescently labelled Nylon 19 × 70 μm; (**j**) PET 17 × 70 μm; (**k**) PP 28 × 100 μm; images taken at x1000 magnification (5 kV, Jeol JSM 6390 LV scanning electron microscope). Photographed by Dr Matthew Cole.

**Figure 2:**
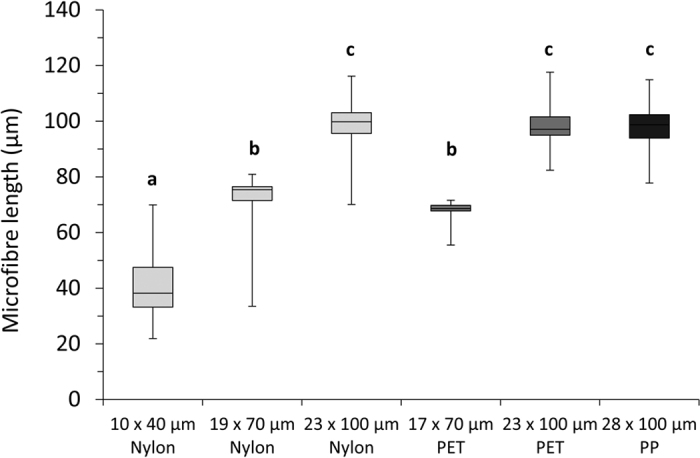
The size of prepared microfibers matched targeted lengths: 40 μm (Nylon 10 μm); 70 μm (Nylon 19 μm; PET 17 μm); 100 μm (Nylon 23 μm; PET 23 μm; PP 28 μm). Box-and-whisker plot shows the full spread of data, including median, inter-quartile and min-max values. Letters denote significant differences in lengths of fibers (ANOVA, Tukey Post-hoc test).

The cryotome protocol proved effective, with the capacity for producing tens of thousands of MFs in <1 hour. Using the prescribed set-up (SI Figure S1), 10 complete spool rotations of polyfilament line (analogous to 3.07 m, of which 2.6 m was sectioned) would yield approximately 58,500 (40 μm), 33,400 (70 μm) or 23,400 (100 μm) MFs, assuming 90% methodological efficiency. The method was applicable for each of the three polymers tested (Fig. 1). MF widths were restricted by the filament diameter of commercially available polyfilament fibers, nonetheless, production of microfibers with 10–28 μm diameters is wholly representative of MF widths observed in the environment^13^. Prepared MFs closely matched their intended lengths (40, 70 or 100 μm; Fig. 2). The greatest degree of size variation was observed in 10 × 40 μm and 19 × 70 μm Nylon microfibers, with standard deviations of 12.4 and 11.1 μm respectively. This variation can be attributed to the elasticity of the polymers and misalignment of synthetic filaments in polyfilament bundles. There were significant differences between the lengths of MFs cut to 40, 70 and 100 μm (ANOVA, *P* < 0.05; Fig. 2). Should narrower size distributions be required MFs could be size fractionated using sieves, per the protocol of Watts, *et al.*^37^. Owing to the constraints of the cryogenic microtome, the maximum length for microfibers is 100 μm; for fibers >100 μm in length, cryogenic grinding would be the recommended method.

A key advantage of the cryotome protocol is its versatility. Recent experiments with Pacific oyster larvae (*Crassostrea gigas*) and polystyrene microspheres (0.7–20 μm) demonstrated microplastic uptake is size dependent^30^. It is therefore essential that researchers can adapt the technique to produce MFs with dimensions most appropriate to their test organism. For example, exposure studies centred around suspension feeding invertebrates (e.g. zooplankton, bivalves) may use microfibers with 40–70 μm lengths, to mimic the shape and size of chain forming centric diatoms (e.g. *Chaetoceros* sp., *Skeletonema* sp.)^38^. Here, fibres were prepared to a minimum 1:3 aspect ratio to demonstrate the applicability of the method; different aspect ratios can be achieved by cutting fibres to longer (e.g. sectioning 10 μm filaments to100 μm lengths to attain MFs with a 1:10 aspect ratio) or shorter lengths, for example: sectioning 100 μm diameter Nylon monofilament line into 10 μm lengths to produce microplastic discs (SI, Figure S2). Embedding and sectioning pre-aligned fibrous materials (e.g. rope) is also possible, however the complex structure and binding agents used in these materials tends to result in the production of microfibers inconsistent in shape and size (personal observations). The cost-effectiveness of the protocol will largely depend on resource availability (i.e. −80 °C freezer, cryogenic microtome) and researcher time; consumables (e.g. freezing solution, spool manufacture) were relatively inexpensive, and, at the time of writing, polymeric fibres cost £81-110 GBP ($106-144 USD) per 200 m reel.

**Figure 3:**
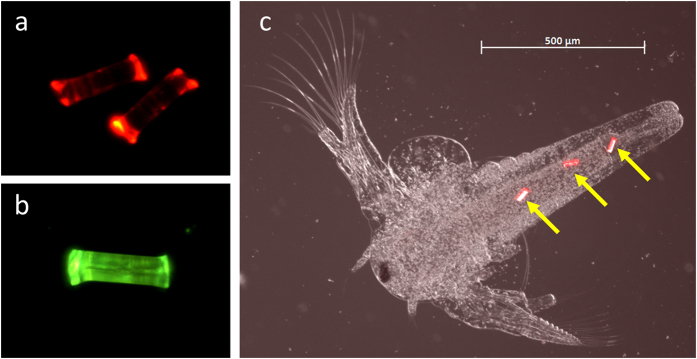
Microplastic fibers were fluorescently dyed using Nile Red and successfully incorporated into a microplastic uptake experiment. Micrographs: (**a**) fluorescent PET MFs (23 × 100 μm) at 515–560 nm excitation; (**b**) fluorescent PP MF (28 × 100 μm) at 450–490 nm excitation; (**c**) fluorescent Nylon MFs (10 × 40 μm; yellow arrows) in the intestinal tract of a 50 h.p.f. brine shrimp (*Artemia* sp.), with 515–560 nm fluorescent excitation. Images taken at x25–200 magnification (Zeiss Observer Z1; AxioVision LE). Photographed by Dr Matthew Cole.

Nile Red dye was applied to nylon, PET and PP MFs, which were observed to fluoresce across a broad spectrum (450–560 nm excitation; Fig. 3); further testing has demonstrated Nile Red is also suitable for dying plastic particulates and irregularly shaped microplastics (data not shown). The acetone rinse caused no observable impairment to the microfibers. Until now, uptake studies have largely relied on the use of fluorescently labelled microspheres, purchased from specialist manufactures, to demonstrate the adherence or ingestion of microplastics^22^. It is anticipated that using Nile Red to fluorescently dye microplastics of different shapes will encourage the use of alternate plastic forms in future experimental design.

A proof of principle experiment was used to demonstrate the applicability of cryotome prepared MFs in laboratory based exposures. Following a 2 h exposure, fluorescent Nylon MFs (10 × 40 μm, 100 MFs mL^−1^) were observed in the intestinal tracts of 72 ± 2.2% of the *Artemia* nauplii visualised (Fig. 3); between 0–7 MFs were identified in the guts, with an average load of 1.4 ± 0.1 MFs organism^−1^. This is the first indication that *Artemia* sp. can ingest microplastic fibers. Prior work has demonstrated *Artemia* nauplii can ingest 1–5 μm and 10–20 μm fluorescent PE microbeads (1.2 × 10^6^ microplastics per 20,000 nauplii), which were trophically transferred to predatory zebrafish (*Danio rerio*)^39^. In another study, consumption and adherence of 40 nm NH_2_ and 50 nm COOH coated PS nanoparticles (5–100 μg mL^−1^) resulted in impaired feeding, altered motility and increased molting in *Artemia franciscana* larvae^40^.

At the nanoscale, fibrous particles have been associated with acute toxicological effects not observed with exposures to spherical particulates of the same material. *In vitro* studies have revealed <100 nm diameter carbon nanotubules (CNTs, 10–20 μm lengths) and asbestos fibrils (3–20 μm lengths) cannot be fully encapsulated by macrophages, causing ‘frustrated phagocytosis’; this can result in release of free radicals and cytokines triggering local inflammation^41^,^42^,^43^. In humans, the chronic effects of asbestos inhalation include pleural fibrosis (scarring), malignant mesothelioma (tumour formation) and carcinoma (cancer)^44^. The Fiber Pathogenicity Paradigm determines that high aspect ratio, biopersistence and small size are key attributes in determining the toxicity of fibrous particulates^42^. It remains to be seen whether the high aspect ratio, environmental persistence and small size of synthetic microfibers represents a heightened toxicological threat when compared with their spherical counterparts. With an absence of data, it is imperative that toxicity testing of MFs is prioritised if we hope to gain a better understanding of the health risks environmentally relevant plastics might have on biota.

Under the EU Marine Strategy Framework Directive (MSFD; Task 10, Marine Litter), a key target for member countries is good environment status, whereby “properties and quantities of marine litter do not cause harm to the coastal and marine environment”^19^. To accurately assess the impact of microplastics on biotic health it is imperative that toxicity tests incorporate environmentally relevant microplastics at ecologically relevant concentrations^32^. Microplastic fibers have been documented in marine biota including mussels^15^, zooplankton^16^ and benthic invertebrates^17^, yet the risk these microplastics pose to biotic health are currently unknown. The development of the cryotome protocol and Nile Red dying method is intended to facilitate researchers with a concise, effective and replicable method for integrating microfibers into future work. It is anticipated toxicity tests will elucidate the risk microplastics fibers pose to vulnerable organisms and ecosystems, and ultimately encourage manufactures to better consider the development of synthetic products that are ‘benign-by-design’^45^.

## Materials and Methods

### Plastic fibers

MFs were prepared using reels of transparent, polyfilament synthetic fiber. Fibers were selected to encompass polymers commonly identified in marine samples^9^,^46^ -nylon (polyamide), polyethylene terephthalate (polyester, PET) and polypropylene (PP)-with a range of filament diameters (Table 1). Fiber diameter was confirmed under an inverted light microscope (x5-x40 objective; Leica DMI 4000). In preparing MFs, a minimum of a 3:1 length:diameter aspect ratio was targeted to ensure a “fibrous” particle and demonstrate the applicability of this novel method.

### Novel microfiber preparation protocol

MFs were prepared using a novel ‘cryotome’ method. A step-by-step guide, complete with photographs, is available at: http://www.cincopa.com/media-platform/test?fid=A0KAGfdQP9_T. In summary: Fibers were aligned by wrapping them around a custom manufactured spool (SI Figure S1); loose ends were secured with tape and fibers kept taut throughout. To prevent improper sectioning, fibers with a high filament density (>80 filaments) were limited to <10 rotations. Aligned fibers were coated with a thin layer of a glycol based, water-soluble freezing solution (Neg 50™, Richard-Allan Scientific or Tissue-Tek^®^, Sakura) and then frozen (10 min; −80 °C; New Brunswick U570 ultra low temperature freezer). Fiber orientation was denoted using a highlighter, and then a scalpel used to excise the parallel lengths of fibers. Freezing agent was used to bind the aligned fibers into a singular block, which was subsequently cut into ~10 mm lengths and moulded into a compact block, ensuring all fibers remained in alignment. Fibers were regularly returned to the freezer to prevent the freezing agent softening and fibers becoming misaligned. Freezing agent was used to secure the block of aligned fibers to a cryogenic microtome (cryotome) mount, with the fibers orientated perpendicular to the base. Fibers were sectioned continuously at pre-determined lengths (40–100 μm; LEICA CM1950). Sections were collected in a Pyrex glass beaker and thawed by adding ultrapure water and heating (15 min; 60 °C; Genlab prime). MFs were filtered out of solution (8 μm polycarbonate filter, Millipore), washed with ultrapure water and subsequently transferred to acid-washed glass vessels. MFs were stored dry or suspended in ultrapure water as appropriate. Mixing hydrophobic or buoyant MFs in water was facilitated by the addition of surfactant (e.g. 0.01% Tween). Airborne contamination of samples was minimised by rinsing all equipment prior to use, covering samples wherever possible and maintaining high standards of cleanliness, per the guidance of Lusher *et al.*^47^.

### Fluorescing plastic

For bioimaging purposes MFs were fluorescently labelled using Nile Red. A Nile Red stock solution (500 μg mL^−1^) was prepared by dissolving 100 mg of Nile Red (technical grade, N3013, SigmaAldrich) in 200 ml acetone; stock solution was stored in the dark at 4 °C. MFs and 2.5 mL of Nile Red solution were carefully transferred into 5 mL Eppendorf tubes; tubes were briefly vortexed and then left to stand for 10 minutes. MFs were vacuum filtered onto 8 μm polycarbonate filters (Millipore), rinsed with acetone to remove excess dye, and then washed with copious amounts of ultrapure water. Fluorescent MFs were kept in the dark prior to experimental use.

### Microfiber analysis

MFs were quantified and sized under a light microscope (Leica DMI 4000), with fluorescent excitation (360 nm, 450–490 nm, or 515–560 nm) where appropriate. MFs were enumerated by diluting MF solutions (10% v/v) and conducting counts with a Sedgewick-rafter chamber. For sizing, image analysis (Image J, FIJI) of randomly selected 1 μL aliquots (accounting for approximately 30 MFs per sample) was conducted. Statistically significant (*P* < 0.05) differences in fiber lengths was determined using ANOVA with Tukey’s post-hoc analysis in ‘R’. High-resolution images were acquired using scanning electron microscopy (SEM). In brief: microfibers were sputter-coated with a 10 nm layer of Au/Pd (Quorum, Q150TES) and visualised at x1000 magnification (5 kV, Jeol JSM 6390 LV).

### Microfiber exposure

To evaluate the applicability of prepared microfibers in laboratory studies, a ‘proof-of-principle’ feeding experiment was conducted. Brine shrimp (*Artemia* sp.; 48 h.p.f.), continuously cultured within the university aquaria, were sub-sampled; ~10 individuals mL^−1^ (*n* = 5 per treatment) were placed in 75 mL artificial seawater (15‰, Tropic Marin^®^) within acid-washed glass jars. Treatments consisted: (i) microplastic-free controls; (ii) experimental treatment containing fluorescent nylon microfibers (10 × 40 μm) at 100 microfibers mL^−1^. Microfibre concentrations were ascertained by diluting stocks (10% v/v) and enumerating microplastics in a Sedgewick-rafter counting chamber. Exposure vessels were maintained on a rotating plankton wheel (<5 rpm), to ensure microplastics remained suspended, in a controlled-temperature laboratory (28 °C) for 2 hours. Post-exposure, seawater was filtered through a 40 μm mesh and brine shrimp retained on the mesh were immediately preserved in 4% buffered formaldehyde. Specimens were visualised using a fluorescent-coupled microscope (Leica DMI 4000; 515–560 nm); the proportion of animals containing microfibers and average MF load was calculated.

## Additional Information

**How to cite this article**: Cole, M. A novel method for preparing microplastic fibers. *Sci. Rep.* **6**, 34519; doi: 10.1038/srep34519 (2016).

## Supplementary Material

Supplementary Information

## Figures and Tables

**Table 1:** A range of synthetic fibers were used to prepare microfibers.

POLYMER	# FILAMENTS/FIBER	FILAMENT DIAMETER (μm)	TARGETTED MF LENGTH (μm)	MANUFACTURER	PRODUCT #
Nylon (6, 6)	14	10	40	Goodfellow	AM325705
Nylon (6, 6)	10	19	70	Goodfellow	AM325720
Nylon (6, 6)	34	23	100	Goodfellow	AM325750
Polyethylene terephthalate	24	17	70	Goodfellow	ES305720
Polyethylene terephthalate	192	23	100	Goodfellow	ES305730
Polypropylene	86	28	100	Goodfellow	PP305747
