# Supplementary material for: A novel method for preparing microplastic fibers
Source: Sci Rep. 2016 Oct 3;6:34519. doi: 10.1038/srep34519 (PMC5046121; doi:10.1038/srep34519)
Supplement: Supplementary Information [file srep34519-s1.pdf]

SUPPLEMENTARY INFORMATION

A novel method for preparing microplastic fibers

Matthew Cole*

College of Life and Environmental Sciences: Biosciences, Geoffrey Pope Building, University of Exeter, Stocker Road, Exeter EX4 4QD, UK

* Corresponding author. Tel.: +44 (0)1392 724677. *E-mail address:* m.cole@exeter.ac.uk

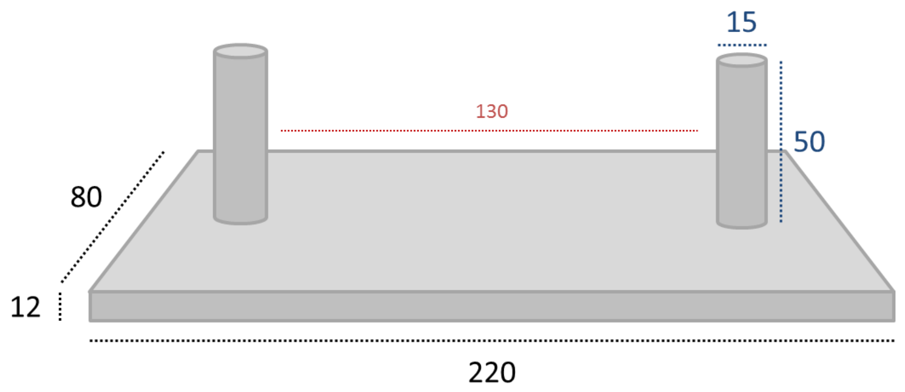

Figure S1. A custom designed 'spool' was manufactured to assist in the rapid alignment of fibres. Dimensions (mm) of the spool can be modified per the requirements of the researcher.

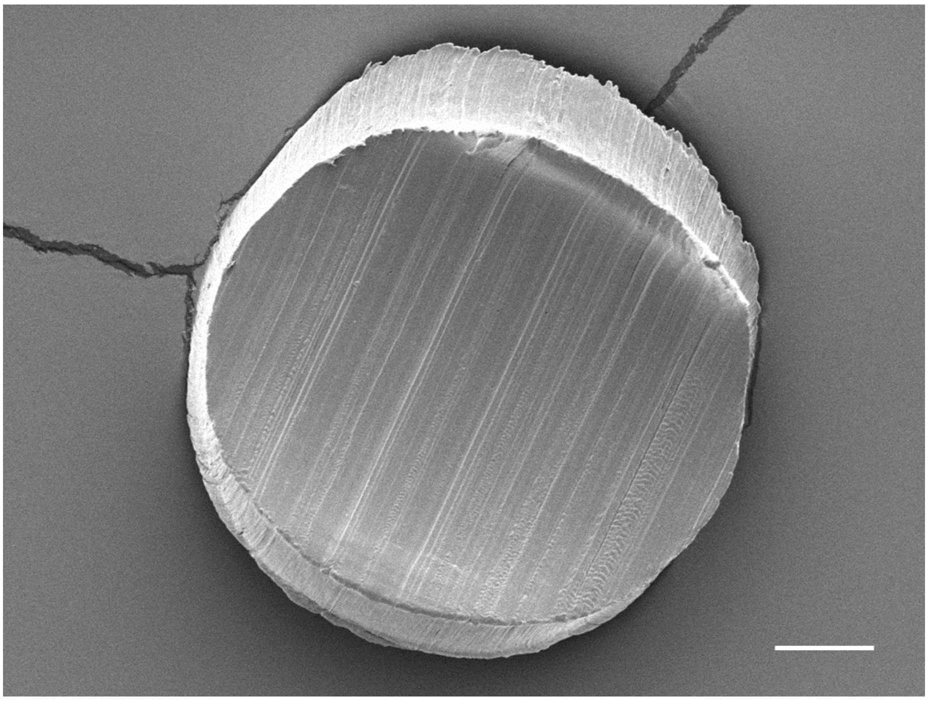

Figure S2. Microplastic 'discs' were prepared by sectioning 100 µm Nylon monofilament fibre into 10 µm lengths. Electron micrograph taken at x700 magnification (5 kV, Jeol JSM 6390 LV scanning electron microscope); white bars: 20 µm. Photographed by Dr Matthew Cole.

Table S1. Laboratory based microplastic research papers published 2000-2015. Research papers identified via Google Scholar using 'microplastic' or 'plastic' search terms. Only laboratory studies considering uptake and/or toxicity of microplastics (<5 mm) were selected. Representative microplastics were categorised as 'spherical microbeads', 'irregularly shaped particles', 'pellets' or 'fibers'. Microplastic polymer (PS, polystyrene; PE, polyethylene; PP, polypropylene; PVC, polyvinyl chloride; CA, cellulose acetate) and size (µm) are given where provided.

STUDY ORGANISM	POLYMER	SIZE (µm)	AUTHOR
**Spherical microbeads: beads, spheres, microspheres (24 studies)**			
Mytilus edulis	PS	3-10	Browne, et al. [1]
Mytilus edulis	PS	0.1	Ward and Kach [2]
Freshwater algae	PS	0.02	Bhattacharya, et al. [3]
Mytilus edulis	PS	0.03	Wegner, et al. [4]
Zooplankton (various)	PS	2-30	Cole, et al. [5]
Mytilus edulis	PS	0.5	Farrell and Nelson [6]
Tripneustes gratilla	PE	32-35	Kaposi, et al. [7]
Tigriopus japonicus	PS	0.05-6	Lee, et al. [8]
Pomatoschistus microps	PE	1-5	Oliveira, et al. [9]
Talitrus saltator	PE	10-45	Ugolini, et al. [10]
Daphnia magna	PS	0.05	Besseling, et al. [11]
Paracentrotus lividus	PS	-	Della Torre, et al. [12]
Idotea emarginata	PS	10	Hämer, et al. [13]
Zooplankton (various)	PS	10	Setälä, et al. [14]
Carcinus maenas	PS	8-10	Watts, et al. [15]
Hyalella azteca	PP	10-27	Au, et al. [16]
Mytilus edulis	PS	0.05	Canesi, et al. [17]
Calanus helgolandicus	PS	20	Cole, et al. [18]
Crassostrea gigas	PS	0.7-20	Cole and Galloway [19]
Tetraselmis chuii	PE	1-5	Davarpanah and Guilhermino [20]
Pomatoschistus microps	PE	420-500	de Sá, et al. [21]
Danio rerio	PE	10-106	Khan, et al. [22]
Marine algae (various)	PS	2	Long, et al. [23]
Pomatoschistus microps	PE	1-5	Luís, et al. [24]
**Irregularly shaped particles: crystals, fluff, powder, granules, fragments, shavings (8 studies)**			
Benthic invertebrates (various)	-	20-2000	Thompson, et al. [25]
Sea cucumbers (various)	PVC	250-1500	Graham and Thompson [26]
Mytilus edulis	PE	80	von Moos, et al. [27]
Arenicola marina	PS	400-1300	Besseling, et al. [28]
Arenicola marina	PVC	230	Browne, et al. [29]
Arenicola marina	PVC	130	Wright, et al. [30]
Idotea emarginata	PS	<100	Hämer, et al. [13]
Dipsastrea pallida	PP	10-2000	Hall, et al. [31]
**Pellets: nurdles, pre-production pellets (4 studies)**			
Sea cucumbers (various)	-	250-1500	Graham and Thompson [26]
Oryzias latipes	PE	3000	Rochman, et al. [32]
Oryzias latipes	PE	3000	Rochman, et al. [33]
Lytechinus variegatus	PE	various	Nobre, et al. [34]
**Fibers: fibers, microfibers, strands, rope (6 studies)**			
Sea cucumbers (various)	PA	250-1500	Graham and Thompson [26]
Nephrops norvegicus	PP	5000	Murray and Cowie [35]
Idotea emarginata	PA	-	Hämer, et al. [13]
Hyalella azteca	PP	20-75	Au, et al. [16]
Carcinus maenas	PP	>500	Watts, et al. [36]
Hediste diversicolor	CA	~120	Wright, et al. [37]

References

1 Browne, M. A., Dissanayake, A., Galloway, T. S., Lowe, D. M. & Thompson, R. C. Ingested Microscopic Plastic Translocates to the Circulatory System of the Mussel, *Mytilus edulis* (L.). *Environmental Science & Technology* **42**, 5026-5031 (2008).

2 Ward, J. E. & Kach, D. J. Marine aggregates facilitate ingestion of nanoparticles by suspension-feeding bivalves. *Marine Environmental Research* **68**, 137-142 (2009).

3 Bhattacharya, P., Lin, S., Turner, J. P. & Ke, P. C. Physical adsorption of charged plastic nanoparticles affects algal photosynthesis. *The Journal of Physical Chemistry C* **114**, 16556-16561 (2010).

4 Wegner, A., Besseling, E., Foekema, E., Kamermans, P. & Koelmans, A. Effects of nanopolystyrene on the feeding behavior of the blue mussel (Mytilus edulis L.). *Environmental Toxicology and Chemistry* **31**, 2490-2497 (2012).

5 Cole, M. *et al.* Microplastic ingestion by zooplankton. *Environmental Science & Technology* **12**, 6646-6655 (2013).

6 Farrell, P. & Nelson, K. Trophic level transfer of microplastic: *Mytilus edulis* (L.) to *Carcinus maenas* (L.). *Environmental Pollution* **177**, 1-3 (2013).

7 Kaposi, K. L., Mos, B., Kelaher, B. & Dworjanyn, S. A. Ingestion of microplastic has limited impact on a marine larva. *Environmental Science & Technology* **48**, 1638-1645 (2013).

8 Lee, K.-W., Shim, W. J., Kwon, O. Y. & Kang, J.-H. Size-Dependent Effects of Micro Polystyrene Particles in the Marine Copepod *Tigriopus japonicus*. *Environmental Science & Technology* **47**, 11278-11283 (2013).

9 Oliveira, M., Ribeiro, A., Hylland, K. & Guilhermino, L. Single and combined effects of microplastics and pyrene on juveniles (0+ group) of the common goby Pomatoschistus microps (Teleostei, Gobiidae). *Ecological Indicators* **34**, 641-647 (2013).

10 Ugolini, A., Ungherese, G., Ciofini, M., Lapucci, A. & Camaiti, M. Microplastic debris in sandhoppers. *Estuarine, Coastal and Shelf Science* **129**, 19-22 (2013).

11 Besseling, E., Wang, B., Lürling, M. & Koelmans, A. A. Nanoplastic affects growth of S. obliquus and reproduction of D. magna. *Environmental science & technology* **48**, 12336-12343 (2014).

12 Della Torre, C. *et al.* Accumulation and Embryotoxicity of Polystyrene Nanoparticles at Early Stage of Development of Sea Urchin Embryos Paracentrotus lividus. *Environmental Science & Technology* **48**, 12302-12311, doi:10.1021/es502569w (2014).

13 Hämer, J., Gutow, L., Köhler, A. & Saborowski, R. Fate of microplastics in the marine isopod Idotea emarginata. *Environmental science & technology* **48**, 13451-13458 (2014).

14 Setälä, O., Fleming-Lehtinen, V. & Lehtiniemi, M. Ingestion and transfer of microplastics in the planktonic food web. *Environmental Pollution* **185**, 77-83 (2014).

15 Watts, A. J. *et al.* Uptake and retention of microplastics by the shore crab Carcinus maenas. *Environmental science & technology* **48**, 8823-8830 (2014).

16 Au, S. Y., Bruce, T. F., Bridges, W. C. & Klaine, S. J. Responses of Hyalella azteca to acute and chronic microplastic. *Environmental Toxicology and Chemistry* (2015).

17 Canesi, L. *et al.* Evidence for immunomodulation and apoptotic processes induced by cationic polystyrene nanoparticles in the hemocytes of the marine bivalve Mytilus. *Marine environmental research* (2015).

18 Cole, M., Lindeque, P., Fileman, E., Halsband, C. & Galloway, T. The impact of polystyrene microplastics on feeding, function and fecundity in the marine copepod *Calanus helgolandicus*. *Environmental Science & Technology* **49**, 1130-1137 (2015).

19 Cole, M. & Galloway, T. Ingestion of nanoplastics and microplastics by Pacific oyster larvae. *Environmental Science & Technology* **49**, 14625–14632 (2015).

20 Davarpanah, E. & Guilhermino, L. Single and combined effects of microplastics and copper on the population growth of the marine microalgae Tetraselmis chuii. *Estuarine, Coastal and Shelf Science*, doi:http://dx.doi.org/10.1016/j.ecss.2015.07.023 (2015).
21 de Sá, L. C., Luís, L. G. & Guilhermino, L. Effects of microplastics on juveniles of the common goby (Pomatoschistus microps): Confusion with prey, reduction of the predatory performance and efficiency, and possible influence of developmental conditions. *Environmental Pollution* **196**, 359-362 (2015).
22 Khan, F. R., Syberg, K., Shashoua, Y. & Bury, N. R. Influence of polyethylene microplastic beads on the uptake and localization of silver in zebrafish (Danio rerio). *Environmental Pollution* **206**, 73-79 (2015).
23 Long, M. *et al.* Interactions between microplastics and phytoplankton aggregates: Impact on their respective fates. *Marine Chemistry* **175**, 39-46 (2015).
24 Luís, L. G., Ferreira, P., Fonte, E., Oliveira, M. & Guilhermino, L. Does the presence of microplastics influence the acute toxicity of chromium (VI) to early juveniles of the common goby (Pomatoschistus microps)? A study with juveniles from two wild estuarine populations. *Aquatic Toxicology* **164**, 163-174 (2015).
25 Thompson, R. C. *et al.* Lost at Sea: Where Is All the Plastic? *Science* **304**, 838, doi:10.1126/science.1094559 (2004).
26 Graham, E. R. & Thompson, J. T. Deposit- and suspension-feeding sea cucumbers (*Echinodermata*) ingest plastic fragments. *Journal of Experimental Marine Biology and Ecology* **368**, 22-29 (2009).
27 von Moos, N., Burkhardt-Holm, P. & Köhler, A. Uptake and effects of microplastics on cells and tissue of the blue mussel *Mytilus edulis* L. after an experimental exposure. *Environmental science & technology* **46**, 11327-11335 (2012).
28 Besseling, E., Wegner, A., Foekema, E. M., van den Heuvel-Greve, M. J. & Koelmans, A. A. Effects of Microplastic on Fitness and PCB Bioaccumulation by the Lugworm Arenicola marina (L.). *Environmental Science & Technology* **47**, 593-600, doi:10.1021/es302763x (2013).
29 Browne, M. A., Niven, S. J., Galloway, T. S., Rowland, S. J. & Thompson, R. C. Microplastic moves pollutants and additives to worms, reducing functions linked to health and biodiversity. *Current Biology* **23**, 2388-2392 (2013).
30 Wright, S. L., Rowe, D., Thompson, R. C. & Galloway, T. S. Microplastic ingestion decreases energy reserves in marine worms. *Current Biology* **23**, 1031-1033 (2013).
31 Hall, N., Berry, K., Rintoul, L. & Hoogenboom, M. Microplastic ingestion by scleractinian corals. *Marine Biology* **162**, 725-732 (2015).
32 Rochman, C. M., Hoh, E., Kurobe, T. & Teh, S. J. Ingested plastic transfers hazardous chemicals to fish and induces hepatic stress. *Scientific reports* **3** (2013).
33 Rochman, C. M., Kurobe, T., Flores, I. & Teh, S. J. Early warning signs of endocrine disruption in adult fish from the ingestion of polyethylene with and without sorbed chemical pollutants from the marine environment. *Science of the Total Environment* **493**, 656-661 (2014).
34 Nobre, C. *et al.* Assessment of microplastic toxicity to embryonic development of the sea urchin Lytechinus variegatus (Echinodermata: Echinoidea). *Marine pollution bulletin* **92**, 99-104 (2015).
35 Murray, F. & Cowie, P. R. Plastic contamination in the decapod crustacean Nephrops norvegicus (Linnaeus, 1758). *Marine Pollution Bulletin* **62**, 1207-1217 (2011).
36 Watts, A. J. R., Urbina, M. A., Corr, S., Lewis, C. & Galloway, T. S. Ingestion of plastic microfibers by the crab Carcinus maenas and its effect on food consumption and energy balance. *Environmental science & technology* (2015).
37 Wright, S. L., Rowe, D., Reid, M. J., Thomas, K. V. & Galloway, T. S. Bioaccumulation and biological effects of cigarette litter in marine worms. *Scientific reports* **5** (2015).
